# Supplementary material for: Effectiveness, tolerability and safety of Direct Acting Antivirals in Mexican individuals with Hepatitis C virus genotype-1 and previous pegylated interferon and ribavirin therapy
Source: PeerJ. 2021 Sep 17;9:e12051. doi: 10.7717/peerj.12051 (PMC8451435; doi:10.7717/peerj.12051)
Supplement: Supplemental Information 1 [file peerj-09-12051-s001.docx]

Table S1. Demographics and clinical characteristics of the 167 non-responder patients to pegIFNα2a+RBV (2005-2013), comparing patients that did not return for further treatment with those that returned for DAA treatment.

|  | Patients that did not return for DAA treatment (84) | Patients that returned  for DAA treatment (83) |
| --- | --- | --- |
| *Sex* |  |  |
| Women, n (%) | 53 (61.9) | 62 (74.6) |
| Men, n (%) | 31 (38.1) | 21 (25.4) |
| *Mean* (95% CI)* |  |  |
| All  Female | 48.4(47.0-49.7)  51.4 (49.9-52.9) | 48.1(46.7-49.9)  51.7 (50.2-53.2) |
| Male | 45.4 (44.2-46.6) | 44.5 (43.3-45.7) |
| *≥ 50 year old*, n (%)* | 41 (50.0) | 45 (54.2) |
| *Cirrhosis*, n (%)* |  |  |
| Yes | 43 (51.1) | 23 (27.7) |
| No | 41 (48.9) | 60 (72.3) |
| *Comorbidity*, n(%)* |  |  |
| Type 2 Diabetes | 16 (19.0) | 9 (10.8) |
| Haemophiliac | 3 (3.6) | 0 (0.00) |
| HIV | 1 (1.19) | 0 (0) |
| HBV | 3 (3.6) | 0 (0.00) |
| *Viral load before pegIFNα2a+RBV treatment (Log_10_ UI/ml)* | | |
| Mean (95% CI) | 5.79 (5.70-5.88) | 5.70 (5.01-5.57) |
| >5.70, n (%) | 41 (48.8) | 44 (53) |
| *Response to pegIFNα2a+RBV (%)* | | |
| Null response | 68 (81) | 55 (66.2) |
| Relapse | 14 (16.6) | 24 (28.9) |
| Breakthrough | 2 (2.4) | 4 (4.8) |
| *Intermediate responses in relapsed patients, n (%)* | | |
| RVR | 3 (21.4) | 7 (29.2) |
| cEVR | 9 (64.3) | 14 (58.3) |
| pEVR | 2 (14.3) | 2 (14.3) |
| SLVR | 0 (0.0) | 1 (4.2) |
| *Developed HCC **, n (%)* | 6 (7.1) | 0 (0.0) |
| *Deaths **, n (%)* | 13 (15.4) | 0 (0.0) |

* At pegIFNα2a+RBV treatment start; ** Until 2014, HCC: hepatocellular carcinoma, HIV: Human immunodeficiency virus, HBV: Hepatitis B virus. *RVR=* rapid virological response, *cEVR=* complete early virological response, *pEVR=* partial early virological response, SLVR= slow virological response.
