# Supplementary material for: Effectiveness, tolerability and safety of Direct Acting Antivirals in Mexican individuals with Hepatitis C virus genotype-1 and previous pegylated interferon and ribavirin therapy
Source: PeerJ. 2021 Sep 17;9:e12051. doi: 10.7717/peerj.12051 (PMC8451435; doi:10.7717/peerj.12051)
Supplement: Supplemental Information 2 [file peerj-09-12051-s002.docx]

Table S2.

Univariate analysis of factors associated to DAA treatment failure with **ombitasvir/paritaprevir/ritonavir/dasabuvir±Ribavirin** (OBV/PTV/r/DSV ±RBV)

| Factor |  | | | |
| --- | --- | --- | --- | --- |
|  | All patients with  *OBV/PTV/r/DSV±RBV*, n | Patients with *OBV/PTV/r/DSV±RBV* and failure, n (%) | RR (95% CI) | *p* |
| *Sex* | | | | |
| Men | 10 | 2 (20) | 0.0 | 0.060 |
| Women | 29 | 0 (0.0) |  |  |
| Age | | | | |
| ≥ 50 years | 34 | 1 (29.4) | 3.33 (0.23-48.1) | 0.413 |
| <50 years | 5 | 1 (20) | 6.8 (0.5-92) | 0.242 |
| *METAVIR score* | | | | |
| F0-F3 | 21 | 0 (0.0) | 0.0 | 0.231 |
| F4 | 18 | 2 (9.5) |  |  |
| Viral subtype |  |  |  |  |
| 1a | 8 | 1 (25) | 3.8 (0.3-55) | 0.372 |
| 1b | 31 | 1 (32.2) | 2.5 (0.17-37) | 0.490 |
| *Initial Viral load (*log_10_ IU/mL) | | | | |
| ≥ 5.58* | 15 | 2 (13.3) | 0.0 | 0.184 |
| < 5.58* | 24 | 0 (0.0) |  |  |
| *Null response to pegIFNα2a+RBV* | | | | |
| Yes | 27 | 1 (3.70) | 2.2 (0.15-33) | 0.526 |
| No | 12 | 1 (8.33) | 0.4 (0.03-6.5) | 0.526 |
| Steatosis |  |  |  |  |
| Yes | 23 | 1 (4.34) | 1.43 (0.09-21) | 0.659 |
| No | 16 | 1 (6.25) | 1.43 (0.09-21) | 1.000 |
| Platelet count<100,000/mm^3^ | | | | |
| Yes | 11 | 0 (0.0) | 0.0 | 0.740 |
| No | 28 | 2 (7.14) |  |  |
| Albumin <3.5 g/dL | | | | |
| Yes | 8 | 0 (0.0) | 0.0 | 0.850 |
| No | 31 | 2 (6.45) |  |  |
| Bilirrubin >2.0 mg/dL | | | | |
| Yes | 4 | 0 (0.0) | 0.0 | 0.720 |
| No | 35 | 2 (5.71) |  |  |
| Obesity | | | | |
| Yes | 7 | 0 (0.0) | 0.0 | 1.00 |
| No | 32 | 2 (6.25) |  |  |
| Diabetes mellitus | | | | |
| Yes | 5 | 0 (0.0) | 0.0 | 1.00 |
| No | 34 | 2 (5.88) |  |  |
| Smoking | | | | |
| Yes | 3 | 0 (0.0) | 0.0 | 1.00 |
| No | 36 | 2 (5.55) |  |  |
| Alcohol use disorder | | |  |  |
| Yes | 5 | 0 (0.0) | 0.0 | 1.00 |
| No | 34 | 2 (5.88) |  |  |

*Mean viral load = 5.58 log_10_ IU/mL of the 39 patients with OBV/PTV/r/DSV±RBV treatment.
